# Evaluation of Occupational Exposure to Perchlorethylene in a Group of Italian Dry Cleaners Using Noninvasive Exposure Indices

**DOI:** 10.3390/ijerph16162832

**Published:** 2019-08-08

**Authors:** Alberto Modenese, Tiziana Concetta Gioia, Andrea Chiesi, Carlotta Abbacchini, Lucia Borsari, Davide Ferrari, Fabrizio De Pasquale, Renato Di Rico, Raffaella Ricci, Antonella Sala, Ennio Gianaroli, Guerrino Predieri, Sara Verri, Fabriziomaria Gobba

**Affiliations:** 1 Department of Biomedical, Metabolic and Neural Sciences, University of Modena & Reggio Emilia, 41124 Modena, Italy; 2 Department of Public Health, AUSL, 41126 Modena, Italy; 3 TEST S.r.l., 41121 Modena, Italy

**Keywords:** perchloroethylene, dry cleaners, occupational exposure, chemical exposure, environmental monitoring, biological monitoring

## Abstract

Recent data suggest a general trend in decreased occupational exposure to perchlorethylene (PCE) in the dry-cleaning sector. The aims of this study were to confirm this trend to lower exposure levels in a group of Italian dry cleaners and to evaluate the current occupational PCE exposure in these works using noninvasive biological indices. Environmental exposure was assessed by personal sampling in 60 operators working in 21 dry cleaning shops in North Italy. PCE in the exhaled alveolar air (PCE*alv*), urinary concentration of PCE and of trichloroacetic acid (TCA) (PCE*u* and TCA*u* respectively), were measured as biological exposure indices. Median PCE environmental concentration in the whole sample was 10.6 mg/m^3^ (i.e., less than the 25% of the levels measured in the same area in a previous study). All values were less than 10% of the occupational limits. PCE*u* measured in samples collected at the end of the work shift resulted the biological markers having the strongest correlation with environmental PCE (r = 0.81). PCE*alv* also resulted in a high correlation (r = 0.66), while a lower correlation was found for TCA*u* measured at the end shift (r = 0.32). According to our results, PCE*u* can be proposed as a valid, noninvasive, and easily reliable exposure index to evaluate PCE exposure at the low levels currently observed in dry cleaners, therefore representing a promising alternative to invasive blood sample collections needed to determine PCE blood concentration.

## 1. Introduction

Perchlorethylene (PCE, CAS registry 127-18-4), is a commercially relevant chlorinated hydrocarbon widely used as a solvent and as a chemical intermediate [1,2]. The use of PCE in dry cleaning was introduced during the first half of the 19th century to replace more toxic solvents, such as carbon tetrachloride, and it has quickly become the main solvent used worldwide [3]. The occupational risk related to PCE exposure in dry cleaning workers was rapidly recognized. Accordingly, new technologies and more effective preventive interventions have been developed and applied over time, leading to a significant decrease of occupational exposure levels, even if high exposures have been occasionally reported [1,2,3,4,5,6,7,8].

In dry cleaners the respiratory system represents usually the main route for PCE absorption: pulmonary uptake is rapid, but complete tissue equilibrium occurs only after several hours. Skin and mucosal absorption is also possible, but, in usual conditions, the contribution is considered scarcely significant [1,3]. Studies in both animals and humans show a relatively low metabolism of PCE, especially at higher exposure levels, even if some variability was observed. Regardless of the route of exposure, most of the absorbed dose (>80%) is excreted unchanged in exhaled air [1]. Elimination of the solvent through the respiratory tract has a biphasic course, probably due to the accumulation and subsequent releasing from the adipose tissue [9,10,11]. A small quote of absorbed PCE is excreted in urine mainly in the form of metabolites (<3%), the major of which is trichloroacetic acid (TCA) [1,3,12], while a smaller quote is also excreted unmodified [7,13,14].

Considering the adverse effects, at high concentrations PCE acts as an irritant for the eyes, the upper airways, and the skin, and it can induce acute effects to the central nervous system (CNS) [1,2,15]. On the other hand, especially at the moderate/low levels of exposure currently expected in dry cleaning, the main health problems are related to long-term, adverse effects. PCE exposure has been classified by the International Agency for Research on Cancer (IARC) as “probably carcinogenic to humans” (Group 2A) [3]; positive associations have been observed for bladder cancer and also for other cancer sites such as the esophagus, kidney, cervix, and in non-Hodgkin’s lymphoma, even if overall results were considered inconsistent [3]. Other long-term, adverse effects, mainly to the liver, kidney, and nervous system functions and, possibly, immune function, have also been reported in workers exposed to PCE [1,3,4,16,17]. According to some previously published data, the main target at low exposure levels can be considered the nervous system: a dose-related effect on color perception was observed in dry cleaning operators, also in exposure to PCE concentrations, largely below the limit proposed by the American Conference of Governmental Industrial Hygienists (ACGIH) [6,18,19,20].

The threshold limit value (TLV) time-weighted average (TWA) proposed by ACGIH for PCE is currently 25 ppm (170 mg/m³) [18], while the corresponding EU Occupational Exposure Limit (OEL) is 20 ppm (138 mg/m³) [21], and the German Maximale Arbeitsplatz-Konzentration (MAK) is still lower at 10 ppm (69 mg/m³) [22]. Perchloroethylene absorption and metabolism can vary significantly according to several factors including (besides environmental concentrations) age, sex, energy expenditure and the body percentage of adipose tissue, inter-individual metabolism variability, and other factors [1,3]. Accordingly, in workers exposed to similar PCE environmental concentrations, the absorbed dose and, consequently, the values of the exposure indices can vary significantly [1,2,9,12]. For this reason, the evaluation of PCE exposure and of the occupational risk using biologic indices offers several advantages over environmental indices; this is especially true at low PCE levels where the weight of variability is more relevant. Various indices have been proposed in the past for PCE exposure evaluation as the urinary excretion of the main PCE metabolites, trichloroacetic acid (TCA*u*) and trichloroethanol in urine (TCE*u*), or TCA concentrations in blood (TCA*b*) [10,11]. The biological exposure indices (BEIs) currently proposed by ACGIH are the concentration of PCE in end-exhaled air (PCE*alv*) and in blood (PCE*b*), both in samples collected prior to the start of the shift; the corresponding limits are, respectively, 3 ppm (25 mg/m³) for PCE*alv* and 0.5 mg/L for PCE*b* [18]. For PCE*b* the German Deutsche Forschungsgemeinschaft proposes a Biologische Arbeitsplatztoleranzwer (BAT) value of 200 µg/L) [22]. The concentration of PCE in urine (PCE*u*) has been also proposed as a biological index of exposure [7].

According to Italian legislation, dry cleaning workers undergo regular health surveillance (HS), usually yearly, including exposure evaluation. The application of the exposure indices currently proposed by ACGIH (i.e., PCE*b* and PCE*alv*) for the HS of PCE-exposed workers raises some more practical problems compared to the previously used urinary indices (TCA*u* and TCE*u*); in fact, an appropriate collection of alveolar air samples is relatively more complicated and sensitive, while for PCE*b* an invasive procedure is needed, which is often not easily feasible especially in small dry cleaning shops. For these reasons, TCA*u* is still the exposure index most frequently applied for the HS of dry cleaners, at least in Italy, even though it is not included anymore in the list of BEIs (or of BATs). The problem here is that the correlation of TCA*u* with environmental PCE exposure at work is lower compared to PCE*alv* and PCE*b*, especially at modest levels currently measured; consequently, its reliability for the HS of dry cleaners is also lower [1,4,5,9,10,11,18,23]. On the other hand, in some studies in the past, another noninvasive exposure index, PCE*u*, proved to be highly correlated with environmental concentrations [7,13,14].

The purpose of our study, performed in collaboration with the Occupational Health and Safety Section (SPSAL) of the local Health Service (Azienda USL di Modena), was three-fold: (i) to evaluate the current PCE exposure scenarios in Italian dry cleaning shops of the Modena district, (ii) to test the reliability of TCA*u* as an exposure index for the health surveillance of workers at current PCE levels, and (iii) to evaluate the possibility to apply PCE*u*, the other noninvasive biological index.

## 2. Materials and Methods 

### 2.1. Study Setting

The study was conducted between March and December 2013 within a routine campaign of monitoring of the occupational risks in dry cleaning facilities of the district of Modena (Emilia Romagna Region), promoted by the local Occupational Health and Safety Section of the Department of Public Health of the National Health System (OHS), in full accordance with the Italian national regulations on occupational health and safety as well as in accordance with the principles of the Declaration of Helsinki. Twenty-one dry cleaning shops, considered representative of the whole sample of dry-cleaning facilities of the district, were selected by a group of OHS experts (Davide Ferrari, Fabrizio De Pasquale, Renato Di Rico, Raffaella Ricci, Antonella Sala and Ennio Gianaroli). Six were small shops (up to four workers), and fifteen were larger shops (with more than four workers employed). During worksite inspection, the OHS experts evaluated the environment and work organization. All shops had natural and/or forced ventilation systems. The activities performed in the shops, substantially similar in small and larger shops, were: (i) acceptance and sorting of the garments to be washed, (ii) pre-cleaning spot removal, (iii) cleaning phase in the machines, (iv) extraction of the washed garments from the machines, (v) post-cleaning spot removal, (vi) ironing phase, and (vii) sorting of the garments to return them to the customers. In addition, usually once a day, the dry-cleaning operators had to load the PCE in the machines and recover the sludge. Workers could be engaged mainly, or almost exclusively, in specific tasks such as washing or ironing, or they could be included in all activities.

### 2.2. Study Population 

The dry-cleaning operators included in the study were 18–70 years old. The only inclusion criterion was to have worked full-time in the dry-cleaning facility for at least six months preceding the study. All workers of the selected shops met the criteria. In the twenty-one shops, considered representative of other dry-cleaning facilities of the district of Modena by the OHS experts, 60 operators—25 men (41.7%) and 35 women (58.3%)—were engaged. During the worksite inspection, operators were classified according to the tasks performed as “washers*”* (W; n = 21; i.e., workers engaged in the washing activities), “ironers*”* (I; n = 22; i.e., workers engaged in the ironing activities), and “jolly*”* (J; n = 17; i.e., workers engaged in different tasks, including accepting and delivering the garments, but also washing and ironing when needed. Complete information was given to the workers who were informed of the ongoing exposure evaluation within a routine campaign of monitoring of the occupational risks promoted by the local OHS authority. Workers were then asked whether they would agree on the results being inclusion in a scientific paper, advising that the inclusion was on a voluntary basis and that they would be free to ask for their data to be cancelled at any time. All workers agreed on the inclusion of their data in a scientific paper, and nobody withdrew during the study. Informed consent was also collected from participants.

### 2.3. Method for Occupational Perchlorethylene (PCE) Exposure Assessment

Occupational PCE exposure of the involved dry-cleaning operators was measured during a normal working week on Thursday, considered representative of the other usual working days. In all workers, individual environmental PCE exposure was evaluated using personal samplers, while absorption was estimated using the following noninvasive biological indices: PCE in the exhaled alveolar air (PCE*alv*), PCE in urine (PCE*u*), and trichloroacetic acid in urine (TCA*u*); all the indices were measured in samples collected at the end of the work shift.

The personal passive samplers (SKC 575 Series Passive Samplers for Organic Vapors) were placed for a full work shift (eight hours) on the collar of the worker’s uniform. In order to avoid any possible issues with membrane saturation, the samplers were changed halfway through the work shift (four hours). After collection, the sampler membranes were stored in a refrigerator until the analysis that, in any case, was carried out within three days after the sampling by gas chromatography according to the method previously described elsewhere [4,5,7,14].

Moving now to biological indices, alveolar air sampling for PCE*alv* measurements was performed using 34 cm^3^ one-way glass tubes equipped with two valves according to the same procedure previously reported elsewhere [4,5,14]. Before analysis, performed by direct injection of the sample into the gas chromatograph, the tubes were heated at 37 °C [4,5,14].

In all workers, a sample of urine was collected at the end of the work shift to measure TCA and PCE concentrations. For PCE analysis, urine was transferred just after voiding into 40 mL borosilicate glass vials (Supelchem) with airtight silicone plugs. Just after, another sample was collected for TCAu measurements. All urines samples were kept refrigerated until the analysis, and the concentrations of PCE and TCA were determined within three days by a gas chromatograph (Varian 3380) equipped with a 63Ni electron capture detector as descripted elsewhere [4,5,14], and all data were analyzed using the Chromeleon™ Chromatography Data System (CDS) Software [24]. The detection limit of the method was 0.1 ng of PCE. Precision was calculated from five duplicate determinations on three different standards, 1, 10, and, 100 ng/mL of PCE; coefficients of variation (CV) ranged from 1.5%–4.5%. In the case of the personal dosimeters, PCE adsorbed on the charcoal was desorbed with five milliliters of carbon disulfide and then diluted in *n*-pentane before gas chromatographic analyses that were conducted according to the National Institute of Occupational Safety and Health method [25]. Further details on analytical methods have been previously reported elsewhere [4,5,7,14].

### 2.4. Statistical Analysis

The difference between the groups of operators classified as washers, ironers, and jolly was analyzed by ANOVA and by a *t*-test for continuous variables with normal distribution, while the Kruskal–Wallis and the Mann–Whitney nonparametric tests were applied for non-normally distributed variables. A *p* value <0.01 was adopted as the statistically significant level. The correlation between variables was tested by linear regression analysis: Pearson correlation coefficients were calculated, and the results were confirmed using the Spearman correlation coefficient. If needed, data after square root transformation or 10-base logarithmic transformation were used for the correlations of the indices and for the regression analysis. All the statistical analyzes were performed using the SPSS statistical package for Windows (IBM SPSS Statistics V25.0) [26].

## 3. Results

**Figure 1:**
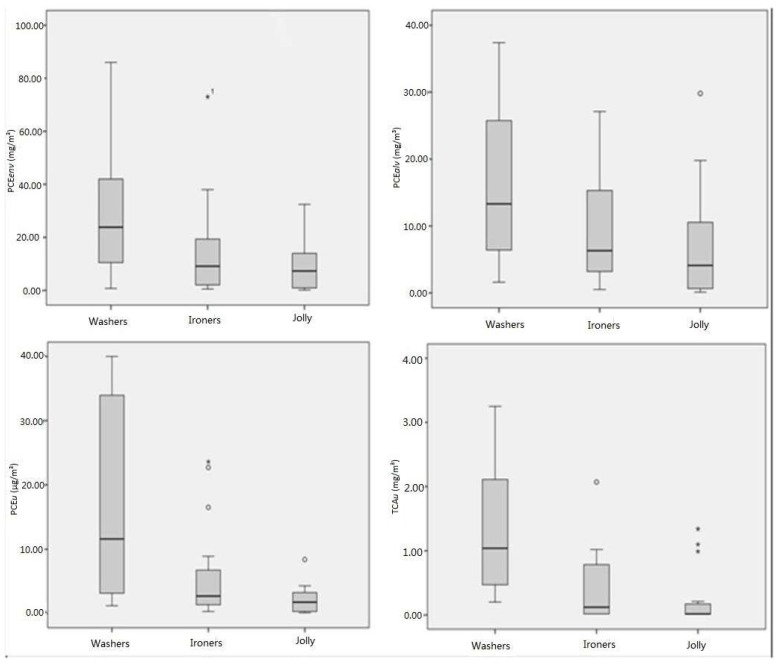
Graphical representation of the occupational perchloroethylene (PCE) exposure indices among the subjects classified according their working tasks (PCE*env*: PCE environmental concentrations, PCE*alv*: PCE in exhaled alveolar air, and PCE and trichloroacetic acid (TCA) in urine samples collected at the end of the work shift (PCE*u* and TCA*u* respectively).^1^ Asterisks and dots in the figure represent outliers.

The results of the individual evaluation of occupational PCE exposure in the examined sample of dry cleaners, including the environmental personal sampling and the biological indices, are reported in Table 1, Table 2 and Table 3 and in Figure 1. In Table 1 we present the values of all exposure indices in the whole sample. In Table 2 and Table 3 and Figure 1 the results obtained in the different occupational groups (“washers”, “ironers”, and “jolly”) are presented: in Table 2 we present the values of environmental PCE concentration (PCE*env*) and end-shift alveolar PCE (PCE*alv*), and in Table 3 the urinary concentrations of PCE (PCE*u*) and of TCA (TCA*u*) at the end of the shift are presented. As the distribution of the values was non-normal, both mean and median values were provided.

Considering the whole sample (Table 1), the mean PCE*env* concentration was 17.0 mg/m³ (SD 18.5), corresponding to 2.5 ppm (i.e., approximately 10% of the TLV-TWA), while the median concentration (10.6 mg/m³) was about 6% of the same limit; the highest environmental value was half of the limit. Considering individual values, 98% of results were lower than half of the TLV-TWA, and 65% were lower than the 10% level. Considering alveolar PCE levels, mean PCE*alv* was 10.4 mg/m^3^, with a maximum value of 37 mg/m^3^. The median value of PCE*u* was 3.1 µg/L (i.e., approximately 6% of the previously estimated Biological Equivalent Exposure Level (BEEL)) [7]. Considering TCA*u* concentrations, the median value was 0.3 mg/L, about 8.5% of the BEI previously proposed by ACGIH [27]. No difference was observed in environmental levels or in biological indices between small and larger shops.

Moving to the specific work tasks, washers were the group with the highest mean exposure (i.e., 27 mg/m³), almost double compared to the 15 mg/m³ of the ironers and more than triple considering the 8 mg/m³ measured for the jolly group (Table 2). Likewise, the results of the other exposure indices were similar: alveolar mean PCE*alv* was 16, 9, and 7 mg/m³ respectively in the washers, ironers, and jolly groups (Table 2), and also mean PCE*u* and mean TCA*u* were significantly higher in washers compared to the other groups (Table 3).

**Figure 2:**
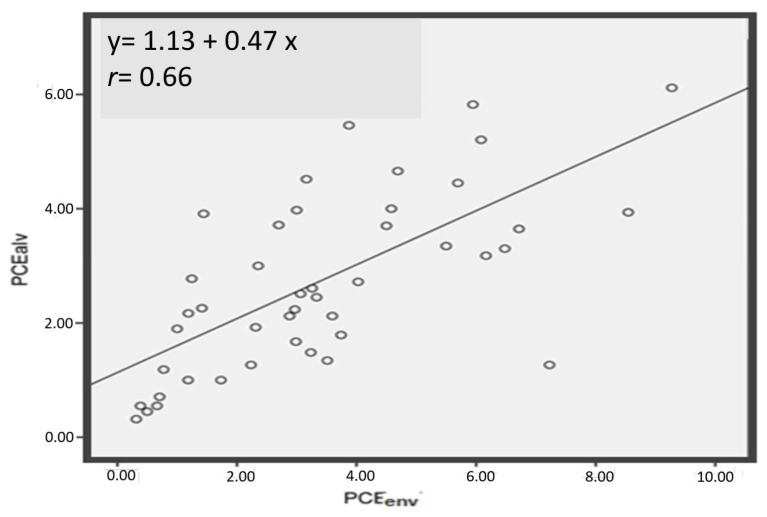
Correlation between environmental PCE concentration in the proximity of workers’ airways (PCE*env*, after square root transformation) and PCE concentration in the exhaled alveolar air at the end of the work shift (PCE*alv*, after square root transformation). The regression line equation and the Spearman r coefficient are also reported.

**Figure 3:**
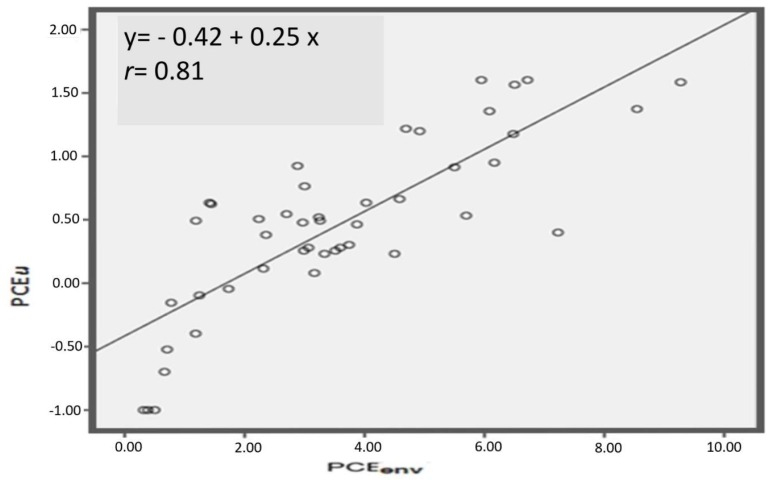
Correlation between environmental PCE concentration in the proximity of workers’ airways (PCE*env*, after square root transformation) and PCE concentration in the urines of the workers at the end of the work shift (PCE*u* after base-10 logarithm transformation). The regression line equation and the Spearman r coefficient are also reported.

**Figure 4:**
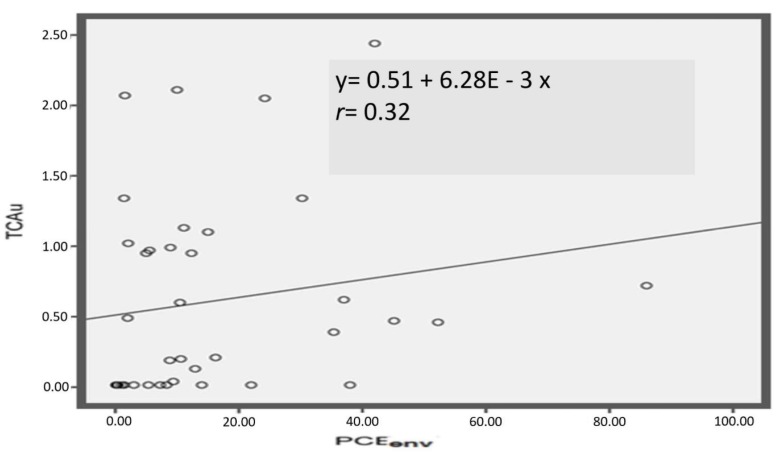
Correlation between PCE concentration in the proximity of workers’ airways (PCE*env*) and TCA concentration in the workers’ urines at the end of the work shift (TCA*u*). The regression line equation and the Spearman r coefficient are also reported.

**Figure 5:**
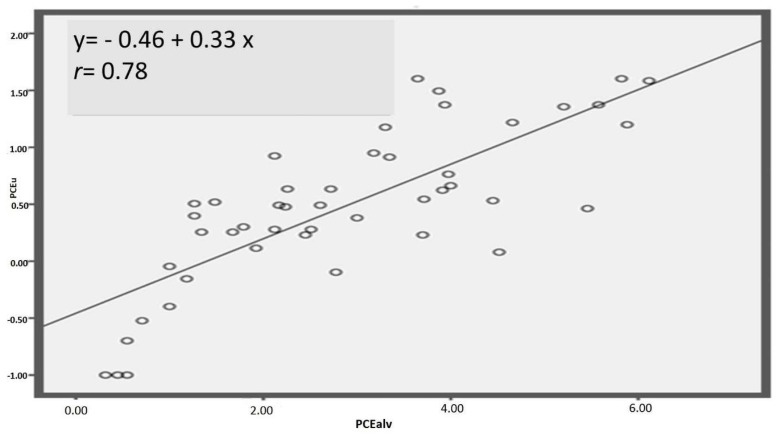
Correlation between PCE concentration in the urines of the workers at the end of the work shift (PCE*u*, after base-10 logarithm transformation) and PCE concentration in the exhaled alveolar air at the end of the work shift (PCE*alv*, after square root transformation). The regression line equation and the Spearman r coefficient are also reported.

The correlations and the regression lines for the different biological exposure indices with environmental PCE concentrations are shown in Figure 2, Figure 3 and Figure 4, and in Figure 5 the correlation between PCE*alv* and PCE*u*, as well as the regression line, are also reported.

In Figure 2 we show the correlation between PCE*env* and PCE*alv* after square root transformation: the correlation coefficient was 0.66. The correlation between PCE*env* after square root transformation and PCE*u* after base-10 logarithmic transformation was higher: *r* = 0.88 (Figure 3). On the other hand, the correlation between PCE*env* and TCA*u* was lower, *r* = 0.32 (Figure 4), and was not improved after transformation of the data (both logarithm and square root transformations). Accordingly, untransformed data for the correlation are presented in Figure 4. Finally, PCE*u* after base-10 logarithmic transformation and PCE*alv* after square root transformation resulted in a correlation coefficient of *r* = 0.78 (Figure 5).

## 4. Discussion

The results obtained showed that PCE exposure in dry cleaning shops in the Modena district, using current technology and prevention, was usually lower than 50% percent of the ACGIH limit (the median levels were lower than 10%) and also lower than the DFG MAK (Table 1 and Figure 1). These values showed a further reduction compared to an investigation in the same area in the early 2000s [14]. They substantially support the decreasing trend of PCE exposure in dry cleaning observed in Europe by other authors [28] who performed a retrospective research in four European Nordic countries (Denmark, Finland, Norway, and Sweden) showing a progressive decrease from 1976 to 2001 in the median PCE environmental concentrations, with values dropping from 136 to 20.4 mg/m³. A similar trend was reported also in USA [3,29,30], and occasionally higher levels have been observed (e.g., in France [31] or in Iran [32]), especially with the occasional possibility of acute effects [33]. In our previous study [14] conducted in a group of dry cleaners of the same territory, we measured median PCE concentrations in the workplaces of 44.2 mg/m³, quite similar to the findings of another North-Italian study [34]. The median PCE concentrations in the exhaled alveolar air of the workers in our previous study was 53.4 mg/m³, while in the current investigation we found environmental and alveolar concentrations of 17.0 and of 10.4 mg/m³ respectively. This decreasing trend is the result of the implementation of new technologies and organizational preventive strategies that improved the working conditions, resulting in low occupational exposure levels [8]. Also at current low PCE levels, task-related differences in individual exposures are clearly valuable: in dry cleaning operators almost/exclusively engaged in washing tasks (washers), environmental PCE levels, even if largely below the proposed occupational limits (as well as the values of biological exposure indices) were about double compared to ironers (Table 2 and Table 3, Figure 1). The lowest PCE exposure (less than 30% compared to washers) was observed in the jolly group having more occasional contacts with the solvent.

As biological exposure indices for the health surveillance of PCE-exposed workers, the ACGIH currently proposes the determination of PCE concentration in end-exhaled air (PCE*alv*) and in blood (PCE*b*), where both samples are collected prior to the start of the shift. However, correct collection of alveolar air for PCE*alv* measurements is more complicated, and sensitive, compared to the collection of urine samples to measure the index previously proposed in dry cleaners (i.e., TCA*u*). On the other hand, an invasive collection of blood is needed for PCE*b*, which is often not easily feasible, especially in the most usual case of collecting samples directly in the shops and, in particular, in small dry-cleaning shops. For these practical reasons, in most cases TCA*u* is the exposure index still routinely used. The problem is that, at current exposure levels, TCA*u* seems not adequately representative of current exposure (Figure 4). So, even if TCA*u* can be considered somewhat representative of group exposure (in fact the values observed in washers, ironers, and jolly groups are different, as reported in Table 3), it seems not adequate at the individual level. This result of the study was in agreement with data previously reported by others [34].

The results obtained support that PCE concentration in end-shift urine is an adequate biological exposure index applicable to evaluate current exposure in dry cleaning workers. Collecting samples to determine the index is noninvasive, and it can be easily performed directly at the workplace and also in small shops (even if an attention must be paid to PCE environmental pollution that, in any case, can be easily checked). Furthermore, the technical procedures for analysis are relatively easy, in any case not more complex compared to PCE*b*, and the analytical method is available in several laboratories. Our results show a good correlation of PCE*u* with environmental personal exposure during the work shift; PCE*u* is also highly correlated with PCE*alv* (Figure 3 and Figure 5).

A relevant point to be considered is that, even if current PCE exposure levels in Italian dry cleaners are quite low compared to the past, research results showed that the risk of side effects, with particular regard to color visual function, could be possible also in the case of an exposure to the solvent significantly below the occupational exposure limits [1,3,6,19,20]. This clearly supports the need to continue regular health surveillance of all exposed dry-cleaning operators and also to consider the case of low exposure levels.

### Study Limitations

This study has some possible limitations. The number of dry-cleaning shops and workers involved is relatively small. On the other hand, shops were selected by an expert group to be representative of other dry-cleaning facilities in the Modena territory. Furthermore, the results are coherent with other similar studies [7,14,17,34]. These points support the representativeness of our sample. Another possible limitation is that we measured exposure during only one day of the working week; however, according to the information collected during the worksite inspections of the OHS experts, the activities performed in dry cleaning were relatively similar during different working days, and we paid attention to follow a “routine” day as similar as possible to the other working days of a normal week. Another point that can be raised here is that we did not apply the biological exposure indices proposed by the ACGIH. On the other hand, the objective of this study was not to compare different exposure indices, rather, as premised, it was limited to evaluating the representativeness of TCA*u* as exposure index at current lower PCE levels in dry cleaning and the possibility to apply PCE*u* as a noninvasive and easily enforceable, practical index.

## 5. Conclusions

The dry-cleaning shops examined in this study were selected to be representative of the facilities of the district of Modena. Furthermore, no difference was observed between small and larger shops. As a consequence, our results show occupational PCE exposure levels in dry cleaners were largely below the exposure limits and were further reduced compared to previous studies. There are no reasons to suppose significant differences in technology and prevention in dry cleaning shops in near geographical areas; accordingly, we are confident that the observed trend and current exposure levels can be considered representative, at least in other parts of North Italy.

At these exposure levels, TCA*u* seems not adequately representative of current exposure at the individual level, even if it still can be representative of group exposure. On the other hand, the noninvasive measurements of PCE concentration in samples of urine collected at the end of the work shift proved a high correlation with PCE exposure measured with individual dosimeters, which was also at low levels. As a conclusion, our findings support the possibility to use PCE*u* as a valid, noninvasive, and easily reliable biological index to evaluate PCE exposure in dry cleaners, even if the low number of subjects involved in our study certainly indicates a need of further research to confirm these findings.

## Figures and Tables

**Table 1:** Results of the evaluation of occupational perchloroethylene (PCE) exposure in the whole sample of dry-cleaning operators. (PCE*env*: PCE environmental concentrations, PCE*alv*: PCE in exhaled alveolar air, and PCE and trichloroacetic acid (TCA) in urine samples collected at the end of the work shift (PCE*u* and TCA*u* respectively).

	PCE_env_ (mg/m³)	PCE_alv_ (mg/m³)	PCE_u_ (µg/L)	TCA_u_ (mg/L)
**Mean (SD) concentration**	17.0 (18.5)	10.4 (10.3)	8.4 (11.7)	0.7 (0.9)
**Median concentration**	10.6	6.6	3.1	0.3
**Range concentration**	0.1–86.0	0.1–37.4	0.1–40.0	0.02–3.2

**Table 2:** Perchloroethylene environmental concentrations measured with personal samplers (PCE*env*), 8 hours TWA, and PCE concentration in end-shift alveolar air (PCE*alv*) in the sample of dry cleaners classified according to their working tasks as “washers”, “ironers” and “jolly”.

	PCE_env_ (mg/m³)			PCE_alv_ (mg/m³)		
	W	I	J	W	I	J
**Mean (SD)**	26.8 (21.2)	14.8 (17.8)	8.2 (9.3)	16.1 (12.6)	8.8 (7.8)	6.7 (8.8)
**Median**	23.8	9.1	6.3	13.3	6.3	4.1
**Range**	0.7–86.0	0.5–73.0	0.0–32.4	1.6–37.4	0.5–27.1	0.1–29.8
*p*	*W vs. I p = 0.06; W vs. J p = 0.004; I vs. J p = 0.23*			*W vs. I p = 0.02; W vs. J p < 0.0001; I vs. J p = 0.05*		

W= *washers*; I = *ironers*; J=*jolly.*

**Table 3:** Results of the evaluation of occupational perchloroethylene (PCE) exposure in the sample of dry-cleaning operators classified according to their working tasks as “washers”, “ironers”, and “jolly”. Data obtained from the end of the work shift urines on PCE concentration (PCE*u*) and trichloroacetic acid concentration (TCA*u*) are reported.

	PCE_u_ (µg/L)			TCA_u_ (mg/L)		
	W	I	J	W	I	J
**Mean (SD)**	16.9 (15.5)	6.2 (7.7)	2.1 (2.2)	1.4 (1.0)	0.4 (0.6)	0.3 (0.5)
**Median**	11.6	2.7	1.8	1.04	0.1	0.01
**Range**	0.2–40.0	0.3–23.6	0.1–8.4	0.2–3.2	0.02–2.1	0.02–1.3
*p*	*W vs. I p = 0.02; W vs. J p = < 0.001; I vs. J p = 0.05*			*W vs. I p = 0.06; W vs. J p < 0.001; I vs. J p = 0.36*		

W= *washers*; I = *ironers*; J = *jolly.*
